# The launch of the *European Clinical Respiratory Journal*, the scientific forum of the Nordic Respiratory Academy

**DOI:** 10.3402/ecrj.v1.24949

**Published:** 2014-06-05

**Authors:** Leif Bjermer, Vibeke Backer, Ronald Dahl, Zuzana Diamant

**Affiliations:** 1 Department of Respiratory Medicine & Allergology, Skåne University Hospital, Lund, Sweden; 2 Bispebjerg Hospital, Copenhagen, Denmark; 3 Department of Respiratory Diseases & Allergy, Aarhus University Hospital, Arhus, Denmark; 4 Department of General Practice, University Medical Center Groningen and QPS-NL, Groningen, The Netherlands

It is with great pleasure that we, the Editors, announce the launch of a new open access respiratory journal: the *European Clinical Respiratory Journal* (*ECRJ*). The journal is closely associated with the Nordic Respiratory Academy (NORA). Previously, NORA was linked to *Clinical Respiratory Journal* (*CRJ*; Wiley), before Wiley decided to move *CRJ* to China.

The overall purpose of *ECRJ* is to provide an international forum where high quality translational studies meet clinical applications. *ECRJ* aims to publish articles or reviews on clinical or scientific respiratory topics or original research with clinical applications, targeting the readership of both (academic) clinicians and respiratory scientists. The Journal will cover the following topics:

1. Epidemiological data or studies.
2. Preventive strategies.
3. Pathophysiology of inflammatory lung disease – identification of potential future biomarkers and treatment targets.
4. Clinical studies: randomized placebo controlled and observational ‘real-life’ trials; translational research.
5. Novel diagnostic methods, including imaging techniques, physiological tests, and biomarkers.
6. Disease and treatment issues for defined populations, including children and the elderly; self-management.
7. Respiratory intensive and critical care.
8. Updates on the status and advances of general and specific disease areas, including asthma, COPD, cough, interstitial lung disease, cystic fibrosis, infectious lung diseases, tuberculosis, bronchiectasis, occupational and environmental lung disease, OSAS.
9. Progress in smoking intervention and cessation methods.

Additionally, the journal will include an educational section presenting manuscripts or letters to the editor aimed at translating basic science into clinically relevant recommendations. Topics will include, but will not be limited to:

1. Translational topics including future targets or biomarkers of disease.
2. Treatment guidelines overview and background discussions.
3. New recommendations.
4. Literature reviews: including year in review, meta analyses.
5. Case reports.
6. Congress proceedings and reports.

All of the productive editors, who previously worked with *CRJ*, have decided to join *ECRJ*. These individuals are active and well-known in their respective fields. Thus, already from the start we have an excellent team of well-experienced people on board. Furthermore, we have been very successful in recruiting excellent international representatives for the different sections, who bring a solid foundation of competence that can guarantee future success.

Our aim is to develop *ERCJ* to steadily become a forum for both clinicians and scientists from NORA and beyond. Based on the rapid flow of high quality submissions so far, the international reputation of the editorial team, and the solid track-record of the previous journal associated with NORA, we believe the Journal is on a fast-track towards being included in PubMed Central. We look forward to your contributions.

*Leif Bjermer* 
Chief Editor Department of Respiratory Medicine & Allergology
Skåne University Hospital
Lund, Sweden *Vibeke Backer* 
Deputy Chief Editor Bispebjerg Hospital
Copenhagen, Denmark *Ronald Dahl* 
Co-Editor Department of Respiratory Diseases & Allergy
Aarhus University Hospital
Arhus, Denmark *Zuzana Diamant* 
Co-Editor Department of Respiratory Medicine & Allergology
Skåne University Hospital, Lund, Sweden
Department of General Practice
University Medical Center Groningen and QPS-NL
Groningen, The Netherlands

